# Osteoporosis: The Renascent Impact of Vertebral Fractures—A Narrative Review of Diagnosis, Risk Stratification, and Integrated Management

**DOI:** 10.3390/jcm15135033

**Published:** 2026-06-28

**Authors:** Mu-Chieh Chi, Kao-Shang Shih, I-Hsin Chen

**Affiliations:** 1 Shin Kong Wu Ho-Su Memorial Hospital, No. 95, Wenchang Rd., Shilin Dist., Taipei 111, Taiwan; m015652@ms.skh.org.tw; 2 Graduate Institute of Biomedical Engineering, National Taiwan University, No. 1, Sec. 4, Roosevelt Rd., Da’an Dist., Taipei 106, Taiwan; 3 Department of Chemical Engineering and Biotechnology, Tatung University, No. 40, Sec. 3, Zhongshan N. Rd., Zhongshan Dist., Taipei 104, Taiwan

**Keywords:** osteoporotic vertebral fracture, vertebral fracture assessment, osteoporosis, fracture cascade, vertebral augmentation, anabolic therapy, fracture liaison service, opportunistic CT screening, artificial intelligence

## Abstract

Osteoporotic vertebral fractures (OVFs) are the most common osteoporotic fracture type and remain substantially underdiagnosed despite their diagnostic, prognostic, and therapeutic importance. This review examines OVFs as sentinel events in osteoporosis, emphasizing their role in diagnosis, fracture-risk stratification, treatment selection, and secondary fracture prevention. This narrative review synthesizes evidence from contemporary clinical practice guidelines, epidemiological studies, randomized controlled trials, systematic reviews, meta-analyses, and selected observational studies addressing imaging and artificial-intelligence-assisted detection. References were selected based on clinical relevance, methodological rigor, and recency, in keeping with the conventions of a focused narrative review rather than a systematic review. OVFs frequently occur without classic osteoporosis-range bone mineral density and often remain clinically silent or incidentally detected. Nevertheless, they independently predict future vertebral, hip, and non-OVFs, particularly during the early post-fracture period. Vertebral fracture assessment on dual-energy X-ray absorptiometry (DXA), opportunistic CT screening, standardized radiology reporting, and AI-assisted detection may improve case finding. Management should combine acute pain control, rehabilitation, appropriate use of vertebral augmentation in selected patients, pharmacological osteoporosis therapy, and structured secondary prevention. Patients with recent or multiple OVFs often meet criteria for high or very-high fracture risk and may benefit from anabolic-first or sequential therapy strategies. Fracture Liaison Services provide a system-level model to close the diagnosis and treatment gap. Recognizing OVFs as actionable markers of skeletal fragility is essential for reducing subsequent fracture burden.

## 1. Introduction

Osteoporosis, characterized by compromised bone strength predisposing individuals to increased fracture risk, has emerged as a major global public health challenge [1]. The condition affects over 200 million people worldwide, with the prevalence expected to increase substantially as populations age. Among osteoporotic fractures, osteoporotic vertebral fractures (OVFs) represent the most common manifestation, yet paradoxically remain the most underdiagnosed and undertreated [2].

The concept of a “global fracture pandemic” has gained recognition in recent years, reflecting the escalating burden of osteoporotic fractures worldwide. OVFs play a pivotal role in this pandemic, not merely as isolated events but as sentinels of systemic bone fragility and harbingers of future fractures [3]. The presence of an OVF increases the risk of subsequent OVFs by approximately five-fold and the risk of hip fractures by two to three-fold.

Despite their clinical significance, OVFs present unique diagnostic challenges. Approximately two-thirds of OVFs are asymptomatic or present with non-specific symptoms, leading to significant underdiagnosis. This “silent epidemic” contributes to delayed treatment initiation, allowing the fracture cascade to progress unchecked. The consequences extend beyond bone health, encompassing reduced quality of life, increased mortality, and substantial healthcare costs [4].

The objective of this comprehensive review is to examine the multifaceted role of OVFs in the diagnosis and management of osteoporosis, with particular emphasis on their contribution to the global fracture pandemic [5]. We explore current understanding of epidemiology, pathophysiology, diagnostic methodologies, and therapeutic strategies, while highlighting emerging technologies and future directions in this critical area of musculoskeletal medicine [6].

Throughout this review, we return to a single organizing principle: the OVF is the clinical entry point to osteoporosis care, and its recognition should trigger a continuous, integrated pathway of diagnosis, fracture-risk stratification, treatment selection, and secondary fracture prevention. The sections that follow are framed around this pathway.

## 2. Materials and Methods

### Review Design and Scope

This article was designed as a focused narrative review rather than a systematic review or meta-analysis. The objective was to synthesize clinically relevant evidence on the role of OVFs in osteoporosis diagnosis, fracture-risk stratification, acute and long-term management, emerging diagnostic technologies, and secondary fracture prevention. The review was structured around clinically actionable themes: epidemiology and burden, pathophysiology and risk factors, diagnostic challenges, OVFs as predictors of future fractures, treatment strategies, emerging diagnostic and therapeutic approaches, and healthcare-system interventions.

A structured literature search was performed to identify relevant publications addressing OVFs and osteoporosis care. PubMed/MEDLINE, Embase, Web of Science, and the Cochrane Library were searched using combinations of the following terms: “vertebral fracture”, “vertebral compression fracture”, “spinal fracture”, “osteoporotic fracture”, “osteoporosis”, “bone mineral density”, “DXA”, “vertebral fracture assessment”, “opportunistic CT screening”, “artificial intelligence”, “fracture risk”, “fracture cascade”, “imminent fracture risk”, “vertebroplasty”, “kyphoplasty”, “bisphosphonate”, “denosumab”, “teriparatide”, “abaloparatide”, “romosozumab”, “fracture liaison service”, and “secondary fracture prevention”. Reference lists of major guidelines, systematic reviews, and key articles were also reviewed to identify additional relevant studies.

Consistent with the conventions of a narrative review. References were selected by the authors based on clinical relevance, methodological rigor, recency, and contribution to the central themes of the review. Priority was given to recent international clinical practice guidelines, high-quality systematic reviews and meta-analyses, and large randomized controlled trials; representative observational and mechanistic studies were also included where they informed the narrative. The review is therefore best characterized as a focused, clinically oriented narrative synthesis rather than a systematic or scoping review [7].

## 3. Epidemiology of Vertebral Fractures

### 3.1. Global Prevalence and Incidence

OVFs affect an estimated 25% of postmenopausal women in developed countries, with prevalence increasing significantly with age. Population-based studies indicate annual incidence rates ranging from 10.7 per 1000 person-years in women aged 50–54 years to 43.8 per 1000 person-years in those over 85 years. Men are also substantially affected, with prevalence rates approximately 60–70% of those observed in women [8,9].

Geographic variations in OVF rates have been documented, with higher rates reported in Scandinavian countries and North America compared to Asian and Latin American populations [10]. However, these differences are narrowing as lifestyle changes and increased longevity affect populations globally. The European Prospective Osteoporosis Study (EPOS) reported prevalence rates of 12.2% in men and 12% in women aged 50–79 years across multiple European centers [9].

### 3.2. Economic and Healthcare Burden

The economic impact of OVFs is substantial and often underestimated. Direct medical costs in the United States exceed $17 billion annually, with indirect costs from lost productivity adding significantly to this burden. In Europe, the total cost of osteoporotic fractures was estimated at €37 billion in 2010, with OVFs accounting for a substantial proportion [5,11,12].

Healthcare utilization following OVFs includes emergency department visits, hospitalizations, outpatient consultations, and long-term care needs. Patients with OVFs demonstrate increased use of pain medications, physical therapy services, and assistive devices. The economic burden extends to caregivers, who often must reduce work hours or cease employment to provide care.

### 3.3. Projected Trends

Demographic projections indicate a dramatic increase in osteoporotic fractures over the coming decades. Global estimates suggest the number of hip fractures alone will increase from 1.66 million in 1990 to 6.26 million by 2050 [13]. OVFs, as the most common osteoporotic fracture type, are expected to increase proportionally. The fastest growth is anticipated in Asia and Latin America, where aging populations are expanding rapidly.

This projected increase has prompted recognition of osteoporotic fractures as an emerging pandemic, requiring coordinated global health responses. Without effective prevention and treatment strategies, the healthcare and economic consequences will become increasingly unsustainable.

## 4. Pathophysiology and Risk Factors

### 4.1. Bone Microarchitecture and Biomechanics

Osteoporosis is characterized by reduced bone mass and microarchitectural deterioration of bone tissue. In the vertebral body, both trabecular and cortical bone contribute to overall strength, with trabecular bone playing a particularly important role. Age-related bone loss preferentially affects horizontal trabeculae, leading to reduced connectivity and increased susceptibility to compression forces.

Biomechanical studies have demonstrated that vertebral strength depends not only on bone mineral density (BMD) but also on geometric factors, trabecular microarchitecture, and cortical thickness. Finite element analysis has revealed that local stress concentrations at weakened trabecular regions can initiate OVFs even under physiological loads [14]. The anterior vertebral body is particularly vulnerable due to higher mechanical loads during forward flexion.

### 4.2. Primary Risk Factors

Age represents the most significant risk factor for OVFs, with exponential increases in incidence after age 60. This age-related increase reflects cumulative bone loss, deteriorating microarchitecture, and increased fall risk. Female sex is associated with higher fracture rates, primarily due to accelerated bone loss following menopause. However, men experience higher mortality rates following OVFs, highlighting the importance of prevention in both sexes [15].

Genetic factors contribute substantially to fracture risk, with heritability estimates ranging from 50 to 80% for BMD and 25 to 35% for fracture risk [16]. Genome-wide association studies have identified multiple loci associated with BMD and fracture susceptibility, including genes involved in osteoblast and osteoclast function. Family history of osteoporotic fractures, particularly maternal hip fracture, is a well-established clinical risk factor.

### 4.3. Secondary Risk Factors

Numerous medical conditions and medications increase OVF risk independently of BMD [17]. Glucocorticoid therapy represents one of the most potent risk factors, with fracture risk increasing rapidly after treatment initiation. Other medications associated with increased fracture risk include proton pump inhibitors, selective serotonin reuptake inhibitors, thiazolidinediones, and aromatase inhibitors.

Endocrine disorders, including hyperthyroidism, hyperparathyroidism, and diabetes mellitus, are associated with altered bone metabolism and increased fracture risk. Type 2 diabetes presents a particular challenge, as patients often have normal or elevated BMD but increased fracture risk due to impaired bone quality [18]. Rheumatoid arthritis, inflammatory bowel disease, and chronic obstructive pulmonary disease are among the inflammatory conditions associated with increased OVF risk [19].

Lifestyle factors significantly influence fracture risk. Low calcium and vitamin D intake, excessive alcohol consumption, smoking, and physical inactivity all contribute to bone loss and increased fracture susceptibility. Low body mass index (BMI < 20 kg/m^2^) is associated with reduced BMD and increased fracture risk, while obesity may provide some protective effect on BMD but is associated with other health complications [20].

## 5. Clinical Presentation and Diagnostic Challenges

Because diagnosis is the first step of the care pathway that frames this review, the clinical and imaging methods discussed in this section are presented not as ends in themselves but as the means of reliably identifying the OVF that initiates fracture-risk stratification, treatment selection, and secondary prevention.

### 5.1. Clinical Manifestations

The clinical presentation of OVFs ranges from asymptomatic to severely debilitating. Acute symptomatic fractures typically present with sudden-onset back pain, often described as sharp or stabbing, localized to the fracture site. Pain may radiate anteriorly around the chest wall or abdomen, occasionally mimicking cardiac or gastrointestinal emergencies. Physical examination may reveal localized tenderness, kyphotic deformity, and height loss [21].

Chronic manifestations of OVFs include persistent back pain, progressive kyphosis, height loss, and restrictive lung disease due to thoracic cage deformity. Multiple OVFs can result in significant functional limitations, including difficulty with activities of daily living, balance impairment, and increased fall risk [22]. Psychological consequences, including depression, anxiety, and reduced self-esteem, are common and often undertreated.

While localized tenderness and percussion pain over the affected vertebra are commonly elicited in clinical practice, their diagnostic sensitivity and specificity for identifying an underlying OVF are limited. Both findings are non-specific, may also occur with degenerative disease or paraspinal muscular pathology, and have been reported with sensitivity and specificity highly dependent on examiner technique and patient body habitus. The clinical examination should therefore be regarded as supportive rather than diagnostic, and dedicated spine imaging remains essential to confirm an OVF and to guide management.

### 5.2. The Silent Epidemic: Asymptomatic Fractures

Approximately two-thirds of OVFs are clinically silent, detected only incidentally on imaging performed for other indications. These asymptomatic fractures carry similar prognostic implications as symptomatic fractures, with comparable increases in subsequent fracture risk and mortality [2]. The high proportion of asymptomatic fractures contributes significantly to underdiagnosis and delayed treatment initiation.

Several factors contribute to the asymptomatic nature of many OVFs. Gradual compression fractures may occur without a discrete traumatic event, allowing time for physiological adaptation. Additionally, OVFs do not always involve neural structures, avoiding radicular symptoms. The lack of symptoms does not diminish the clinical significance of these fractures, as they represent established bone fragility requiring intervention.

### 5.3. Diagnostic Imaging Modalities

Radiography: Conventional radiography remains the primary imaging modality for detecting OVFs. Lateral thoracic and lumbar spine radiographs allow visualization of vertebral body height and shape. The Genant semiquantitative method is widely used to grade OVFs based on height reduction: grade 1 (20–25% reduction), grade 2 (25–40% reduction), and grade 3 (>40% reduction) [23]. However, radiography has limitations, including the two-dimensional representation of three-dimensional structures and interobserver variability in fracture assessment.

Dual-Energy X-ray Absorptiometry (DXA): While primarily used for BMD assessment, DXA provides crucial information for fracture risk assessment. The World Health Organization defines osteoporosis as BMD T-score ≤ −2.5 at the spine, hip, or forearm. However, many OVFs occur in individuals with osteopenia (T-score between −1.0 and −2.5), highlighting the limitations of BMD alone in predicting fracture risk [24]. Vertebral Fracture Assessment (VFA), a lateral spine imaging technique available on modern DXA machines, allows opportunistic screening for prevalent OVFs during routine BMD testing [24,25,26,27].

Computed Tomography (CT): CT provides superior visualization of vertebral anatomy and is particularly useful for distinguishing osteoporotic fractures from pathological fractures or traumatic injuries. Quantitative CT allows the three-dimensional assessment of volumetric BMD and the separate evaluation of trabecular and cortical bone. High-resolution peripheral quantitative CT (HR-pQCT) enables in vivo assessment of bone microarchitecture, though it is currently limited to peripheral skeletal sites [28,29].

Magnetic Resonance Imaging (MRI): MRI is invaluable for determining fracture acuity, excluding malignancy or infection. Acute osteoporotic fractures demonstrate characteristic bone marrow edema on short tau inversion recovery (STIR) or T2-weighted fat-suppressed sequences. MRI is also superior for visualizing spinal cord and nerve root involvement. However, MRI is more expensive and less accessible than radiography, limiting its use for routine screening [30].

Detecting silent vertebral deformity: Because most OVFs are clinically silent, the morphological criteria used to define a vertebral deformity strongly influence whether it is detected and reported. Three complementary approaches are used: the Genant semiquantitative method, which visually grades deformities by the degree of vertebral height loss; quantitative morphometry, which derives anterior, middle, and posterior vertebral heights from six-point digitisation; and the algorithm-based qualitative (ABQ) method, which focuses on endplate depression to separate genuine osteoporotic fractures from non-fracture deformities. Reported prevalence differs substantially between these methods, underscoring the need for standardized case definitions and structured radiological reporting [31]. A recurring difficulty is distinguishing a true osteoporotic deformity from mimics such as Scheuermann disease, degenerative wedging, Schmorl nodes, and congenital variants, a distinction the ABQ method was specifically designed to improve.

For case-finding in asymptomatic individuals, Vertebral Fracture Assessment (VFA) on modern DXA densitometers provides low-radiation lateral spine imaging at the time of bone-density testing and shows good agreement with spine radiography for moderate-to-severe deformities, although sensitivity is lower for grade 1 deformities and the upper thoracic spine [26,27]. Restricting VFA to defined high-risk indications preserves its diagnostic yield. In parallel, opportunistic review of CT scans obtained for unrelated indications can identify prevalent OVFs at no additional cost or radiation, using low vertebral attenuation at L1 (for example, below approximately 110–135 Hounsfield units) as a practical trigger, increasingly supported by automated and AI-based pipelines [32,33].

### 5.4. Differential Diagnosis

OVFs must be distinguished from other causes of vertebral deformity and back pain. Pathological fractures due to malignancy (primary or metastatic) typically demonstrate cortical destruction, soft tissue masses, and the involvement of posterior elements. Infectious processes, including osteomyelitis and diskitis, present with endplate irregularity, disc space narrowing, and paravertebral soft tissue changes.

Schmorl’s nodes, representing the herniation of disk material through vertebral endplates, can mimic fractures on radiographs but are typically asymptomatic and unassociated with height loss. Degenerative changes, including osteophytes and facet joint arthropathy, may complicate radiographic interpretation. Advanced imaging, particularly MRI, is often necessary to establish accurate diagnosis in ambiguous cases.

### 5.5. The Underdiagnosis Problem

Despite their prevalence and clinical significance, OVFs remain significantly underdiagnosed and underreported [2]. Studies indicate that only 30–35% of radiographically apparent OVFs are mentioned in radiology reports. Even when reported, OVFs often do not trigger appropriate clinical follow-up or osteoporosis evaluation [34].

The underdiagnosis of OVFs is not solely a matter of patient presentation; it reflects the interaction of nonspecific symptoms, inconsistent imaging triggers, variable radiological definitions, and incomplete linkage between imaging findings and osteoporosis care. Table S1 summarizes representative evidence supporting complementary case-finding strategies, including DXA-based vertebral fracture assessment, risk-triggered screening, standardized radiological assessment, opportunistic CT review, and AI-assisted detection.

The practical implication is not that every patient requires every imaging modality. Rather, vertebral-fracture detection should be embedded within a tiered workflow: clinical risk factors should trigger VFA or spine imaging when appropriate; CT scans obtained for unrelated indications should be reviewed opportunistically; and AI-assisted tools may serve as workflow adjuncts to reduce missed fractures. Importantly, detection only improves outcomes if the radiological finding is linked to osteoporosis assessment, fracture-risk stratification, and secondary prevention.

## 6. Vertebral Fractures as Predictors of Future Fractures

### 6.1. The Fracture Cascade Concept

The “fracture cascade” describes the phenomenon whereby an initial fracture substantially increases the risk of subsequent fractures. OVFs are particularly potent predictors of future fractures, both at vertebral and non-vertebral sites [35]. This increased risk reflects underlying bone fragility, altered biomechanics, and often inadequate treatment of the underlying osteoporosis.

The risk of subsequent fractures is highest in the period immediately following an initial OVF. Studies demonstrate that approximately 20% of patients with acute OVFs experience another OVF within one year. This “imminent risk” period represents a critical window for intervention, during which fracture risk can be substantially reduced through appropriate treatment [36].

### 6.2. Risk of Subsequent Vertebral Fractures

Prevalent OVFs increase the risk of future OVFs by approximately five-fold. This risk is independent of BMD, emphasizing that fracture history provides additional prognostic information beyond bone density measurements. The number of prevalent fractures and their severity (as assessed by height reduction) further stratify risk, with multiple or severe fractures conferring particularly high subsequent fracture risk [35].

Biomechanical studies have elucidated mechanisms underlying the increased risk of adjacent OVFs following initial fracture. Vertebral compression alters spinal alignment and load distribution, increasing stress on adjacent vertebrae. Kyphotic deformity shifts the center of gravity anteriorly, creating additional flexion moments on the spine. These biomechanical alterations, combined with systemic bone fragility, create a vicious cycle of progressive fractures.

### 6.3. Risk of Hip and Other Peripheral Fractures

OVFs also predict fractures at non-vertebral sites, particularly hip fractures. Meta-analyses indicate that prevalent OVFs increase hip fracture risk by 2–3 fold [14,16,37]. This association persists after adjustment for BMD and age, suggesting that OVFs identify individuals with impaired bone quality not fully captured by densitometry [18].

The relationship between vertebral and peripheral fractures likely reflects shared risk factors, including systemic bone fragility, propensity to fall, and often delayed or absent treatment. Additionally, functional limitations and balance impairment resulting from OVFs may increase fall risk. Kyphotic posture alters the center of mass, potentially affecting balance and increasing fall susceptibility.

### 6.4. Temporal Patterns of Fracture Risk

Fracture risk following an initial OVF is not constant over time [10,38]. The concept of “imminent fracture risk” recognizes that fracture risk is highest in the first 1–2 years following an incident fracture. During this period, the risk of subsequent OVF may be as high as 20–25%, compared to 3–5% annual risk in untreated patients with prevalent fractures [36].

This temporal pattern has important implications for treatment strategies. Interventions initiated during the imminent risk period have the greatest potential to prevent subsequent fractures. Conversely, delayed treatment initiation allows the fracture cascade to progress, potentially limiting the effectiveness of subsequent interventions. Recognition of this time-dependent risk has led to increased emphasis on rapid osteoporosis assessment and treatment initiation following OVFs [5].

Once an OVF is identified, the clinical question shifts from whether osteoporosis is present to how urgently and intensively fracture risk should be managed. OVFs are clinically important because they integrate several dimensions of risk: established skeletal fragility, future vertebral and non-OVF probability, early post-fracture vulnerability, functional decline, and treatment urgency. Table S2 summarizes the main clinical implications of OVFs as sentinel risk markers.

This evidence supports a vertebral-fracture-centered approach to osteoporosis care. In this framework, BMD remains essential but is not sufficient. OVF status, including fracture number, severity, and recency, should be incorporated into fracture-risk stratification and treatment selection. This approach is particularly relevant for patients with recent or multiple OVFs, who may warrant rapid pharmacological intervention and structured secondary prevention.

### 6.5. Mortality Implications

OVFs are associated with significantly increased mortality risk. Meta-analyses indicate that prevalent OVFs increase all-cause mortality by approximately 30–40% in women and 60–70% in men [39]. This excess mortality persists for several years following fracture and is observed even in asymptomatic fractures detected radiographically.

Multiple mechanisms contribute to increased mortality following OVFs. Direct effects include pulmonary complications related to thoracic cage deformity and restrictive lung disease. Indirect effects include immobility-related complications, including pneumonia, thromboembolic disease, and muscle deconditioning. Additionally, OVFs may serve as markers of general frailty and comorbid conditions. Pain and functional limitations may lead to social isolation, depression, and reduced quality of life, further contributing to adverse outcomes.

## 7. Current Management Strategies

Management follows directly from risk stratification: the more recent or severe the OVF, the stronger the rationale for prompt pharmacological therapy and coordinated secondary prevention, with vertebral augmentation reserved for selected patients with refractory pain. The interventions discussed below are therefore presented as components of a single, integrated care pathway rather than as isolated treatments.

### 7.1. Acute Management

#### 7.1.1. Pain Control

Effective pain management is crucial in the acute phase following OVF. Multimodal analgesia, combining acetaminophen, nonsteroidal anti-inflammatory drugs (NSAIDs), and judicious use of opioids, is recommended. NSAIDs should be used cautiously in elderly patients due to cardiovascular and gastrointestinal risks. Opioids, while effective for severe pain, carry risks of dependence, falls, and cognitive impairment, particularly in older adults.

Calcitonin, traditionally used for pain management in acute OVFs, has shown modest efficacy in some studies, though its use has declined following concerns about malignancy risk with long-term use [40]. Muscle relaxants may provide short-term benefit but should be prescribed cautiously due to side effects.

#### 7.1.2. Conservative Treatment

The majority of acute OVFs are managed conservatively with a combination of pain control, activity modification, and bracing [41]. Controlled mobility with gradual return to activities is preferred over prolonged bed rest, which accelerates bone loss and muscle deconditioning. Hyperextension bracing (thoracolumbosacral orthoses) may provide pain relief and support spinal alignment, though evidence for efficacy is limited and patient acceptance is variable.

#### 7.1.3. Surgical Interventions 

Vertebroplasty and balloon kyphoplasty represent minimally invasive procedures for treating painful OVFs [11,42]. Both involve percutaneous injection of bone cement (polymethylmethacrylate) into the fractured vertebra to provide mechanical stabilization and pain relief.

Vertebroplasty involves direct injection of cement into the vertebral body. Multiple randomized controlled trials have produced conflicting results regarding its efficacy compared to sham procedures. However, these trials have been criticized for methodological limitations, including delayed enrollment and high placebo response rates. Observational studies and registry data generally support the effectiveness of vertebroplasty for pain relief in appropriately selected patients [43,44,45,46,47].

Recent randomized data, however, have produced contradictory conclusions, and the role of vertebral augmentation remains debated. Sham-controlled trials such as the studies by Buchbinder and colleagues (INVEST) and Kallmes and colleagues, both published in 2009, reported no superiority of vertebroplasty over a simulated procedure for pain reduction in chronic or subacute fractures. Subsequent positive trials, including the VAPOUR trial in acute severely painful fractures of less than six weeks duration and the VERTOS II study, suggested meaningful pain and function benefit when patient selection emphasized acute, well-defined, severely painful fractures [14,43,48].

Critiques of the negative sham trials have focused on the enrolment of patients with substantially older fractures and lower pain intensity than typical clinical candidates, low recruitment rates, and high crossover, which may have biased the effect estimates toward the null. The most recent Cochrane review and several meta-analyses therefore emphasize that conclusions depend heavily on the time from fracture onset and pain severity at enrolment [49,50,51].

International clinical guidelines reflect this uncertainty. The National Institute for Health and Care Excellence (NICE) supports vertebroplasty and kyphoplasty in patients with severe ongoing pain after recent OVF and after non-surgical management has failed, whereas the American Academy of Orthopedic Surgeons (AAOS) has historically given a weak or conditional recommendation, and the Society of Interventional Radiology (SIR) endorses vertebral augmentation in carefully selected patients with acute fractures and refractory pain. The lack of guideline convergence reinforces the need for individualized decision-making [22,52,53,54].

Beyond efficacy on pain, long-term outcomes of vertebral augmentation remain incompletely defined. Concerns persist regarding adjacent-level fractures, with several observational series suggesting an increased incidence in cement-augmented vertebrae, although prospective comparative data are inconsistent and confounded by underlying skeletal fragility. Routine pharmacological treatment of osteoporosis after augmentation is essential to address the underlying fragility and to mitigate adjacent-level fracture risk [55,56,57,58,59].

Kyphoplasty involves creating a cavity within the vertebral body using an inflatable balloon before cement injection, potentially allowing better vertebral height restoration and reducing cement leakage risk [60,61]. Randomized trials have demonstrated superior pain relief and functional improvement with kyphoplasty compared to conservative management. A meta-analysis suggested kyphoplasty may provide better height restoration than vertebroplasty, though clinical significance remains debated [62,63,64,65].

Both procedures carry risks, including cement leakage (symptomatic in 1–2% of cases), infection, and possible increased risk of adjacent OVFs [42,61]. Patient selection is crucial, with optimal candidates being those with severe pain refractory to conservative management, recent fracture onset (typically <6 months), and absence of contraindications [66,67].

### 7.2. Pharmacological Treatment for Osteoporosis

#### 7.2.1. Antiresorptive Agents

Bisphosphonates remain the most commonly prescribed medications for osteoporosis treatment [68]. These agents inhibit osteoclast-mediated bone resorption, leading to increased BMD and reduced fracture risk. Oral bisphosphonates (alendronate, risedronate, ibandronate) reduce OVF risk by approximately 40–70% [69,70]. Zoledronic acid, an intravenous bisphosphonate administered annually, has demonstrated similar efficacy with potentially superior adherence.

The optimal duration of bisphosphonate therapy remains debated, with concerns about long-term use including atypical femoral fractures and osteonecrosis of the jaw [71]. Current guidelines suggest drug holidays after 3–5 years of oral bisphosphonates or 3 years of zoledronic acid in patients at moderate fracture risk, with continuation in high-risk patients [19,72].

Denosumab, a fully human monoclonal antibody against RANK ligand, provides potent inhibition of bone resorption. The FREEDOM trial demonstrated 68% reduction in OVF risk with denosumab [69,70]. Denosumab is administered subcutaneously every six months and does not accumulate in bone, allowing more reversible effects than bisphosphonates. However, discontinuation of denosumab results in rapid bone loss and increased fracture risk, necessitating transition to alternative therapy.

Selective estrogen receptor modulators (SERMs), particularly raloxifene, reduce OVF risk by approximately 30–50% but do not significantly reduce non-OVF risk [73,74]. Their use is limited by vasomotor symptoms and increased risk of venous thromboembolism.

#### 7.2.2. Anabolic Agents 

Parathyroid hormone analogs (teriparatide and abaloparatide) stimulate bone formation when administered intermittently. The Fracture Prevention Trial demonstrated 65% reduction in OVFs and 53% reduction in non-vertebral fragility fractures with teriparatide [35,74,75,76,77]. Abaloparatide showed similar efficacy with possibly lower hypercalcemia risk.

Romosozumab, a monoclonal antibody against sclerostin, represents a novel dual-action agent with both bone-forming and antiresorptive properties. The FRAME trial demonstrated 73% reduction in OVFs after one year of romosozumab [78,79]. The ARCH trial showed superior fracture reduction with romosozumab followed by alendronate compared to alendronate alone. However, romosozumab carries a cardiovascular safety warning and is contraindicated in patients with recent myocardial infarction or stroke [80,81].

Anabolic agents are typically limited to 1–2 years of treatment due to efficacy plateaus and regulatory restrictions. Following anabolic therapy, transition to antiresorptive therapy is essential to maintain BMD gains [5,17,19,68,82].

Several practical considerations limit the broad adoption of anabolic-first strategies. Daily subcutaneous teriparatide or abaloparatide and monthly subcutaneous romosozumab are substantially more expensive than oral bisphosphonates, with monthly drug costs typically one to two orders of magnitude higher in many healthcare systems. Reimbursement criteria for these agents vary widely between countries; in many systems anabolic therapy is restricted to patients with very severe osteoporosis or with documented failure of antiresorptive treatment, which can delay access for patients with recent OVF who would benefit from early anabolic intervention. Persistence and adherence to injectable anabolic regimens are influenced by injection burden, side-effect profile, and out-of-pocket cost, and have been shown to be suboptimal in real-world studies. These considerations should be discussed with patients when selecting initial therapy [17,68,82,83,84,85,86].

Combination and Sequential Therapy: Growing evidence supports sequential therapy strategies, particularly anabolic agents followed by antiresorptives [87]. This approach capitalizes on the bone-building effects of anabolic agents while maintaining gains with antiresorptive therapy [68,87]. The DATA study demonstrated superior BMD gains with sequential therapy compared to either agent alone.

Starting with antiresorptive agents may blunt the response to subsequent anabolic therapy, suggesting anabolic-first strategies may be optimal for patients with severe osteoporosis [68,83]. However, practical considerations, including cost and insurance coverage, often necessitate bisphosphonate first-line therapy [88,89,90].

### 7.3. Rehabilitation and Physical Therapy

Rehabilitation plays a crucial role in recovery following OVFs. Physical therapy programs typically progress through phases, beginning with gentle range-of-motion exercises and advancing to strengthening and functional activities. Core strengthening exercises improve trunk stability and may reduce future fracture risk [91].

Balance training is particularly important given the increased fall risk associated with OVFs. Tai Chi and other balance-focused exercises have demonstrated efficacy in reducing falls in osteoporotic populations. Postural training helps counteract kyphotic deformity and may reduce spinal loading [2].

Aerobic exercise, particularly weight-bearing activities, promotes bone health and cardiovascular fitness. Swimming and aquatic therapy provide exercise options for patients unable to tolerate land-based weight-bearing activities. Resistance training stimulates bone formation and improves muscle strength, both important for fracture prevention [92].

### 7.4. Integrated Care Pathway After Vertebral Fracture

Management after OVF should be organized as a care pathway rather than a collection of isolated interventions. Acute pain control, mobilization, rehabilitation, vertebral augmentation, pharmacological therapy, and secondary fracture prevention each address different consequences of the same sentinel event. Table S3 summarizes evidence-informed actions after vertebral-fracture identification and highlights the clinical role and limitations of each intervention.

The central management principle is that vertebral augmentation, pharmacological therapy, rehabilitation, and FLS are not competing strategies. Vertebral augmentation may address persistent fracture-related pain in selected patients, whereas osteoporosis medication and FLS address the underlying skeletal fragility and future fracture risk. Rehabilitation and fall-risk reduction support functional recovery and reduce downstream vulnerability. Therefore, the optimal response to an OVF is integrated, risk-stratified, and longitudinal.

## 8. Emerging and Individualized Approaches

### 8.1. Advanced Imaging Techniques

The detection technologies discussed here are considered specifically as tools to close the diagnostic gap that currently delays entry into this care pathway; the technical detail is therefore kept subordinate to this clinical purpose.

AI and machine-learning applications are increasingly being evaluated as adjuncts for OVF detection and risk prediction. Deep learning algorithms trained on large radiographic datasets can identify OVFs with accuracy comparable to or exceeding expert radiologists [93]. AI-assisted fracture detection systems can flag potential fractures for radiologist review, potentially reducing underdiagnosis [94,95,96,97,98,99].

Opportunistic screening using CT scans performed for other indications represents an underutilized opportunity for OVF detection [29,32,100,101,102,103]. Automated vertebral segmentation and fracture detection algorithms applied to routine chest or abdominal CT scans can identify prevalent OVFs without additional radiation exposure or cost [29,100]. Implementation of such systems could significantly improve OVF detection rates.

Despite encouraging diagnostic-accuracy estimates, several important limitations of AI-assisted vertebral-fracture detection must be acknowledged. External validation across diverse populations, imaging vendors, acquisition protocols, and patient-positioning practices is currently limited; most published deep-learning models have been trained and tested on relatively homogeneous datasets, raising concerns about dataset bias and generalizability. Subgroup performance across age, sex, race, body habitus, and underlying spinal pathology is rarely reported [104,105,106].

False-positive and false-negative outputs carry direct clinical consequences. False positives may trigger unnecessary further imaging, anxiety, and inappropriate pharmacological treatment, whereas false negatives may reinforce existing underdiagnosis of OVFs, particularly in mild grade-1 deformities and in patients with degenerative endplate changes. Alert fatigue is a recognized risk when AI flags are integrated into reporting workflows without careful thresholding [18,94].

Integration into routine clinical workflows raises additional implementation challenges, including interoperability with picture archiving and communication systems (PACS) and reporting platforms, regulatory considerations such as Food and Drug Administration (FDA) and Conformité Européenne (CE) clearance, and the medicolegal question of responsibility when an AI-flagged or AI-missed fracture results in patient harm. AI tools should therefore be deployed as decision support rather than autonomous diagnostic systems, with the responsible radiologist or clinician retaining final interpretive responsibility [6,33,94,107,108].

### 8.2. Bone Quality Assessment Beyond BMD

Recognition that BMD alone inadequately predicts fracture risk has driven development of techniques assessing bone quality [109,110,111]. Trabecular bone score (TBS), derived from DXA images, provides an indirect measure of bone microarchitecture. Lower TBS values predict fracture risk independently of BMD. Integration of TBS with fracture risk assessment tools improves fracture prediction [5,6,14,28].

Quantitative ultrasound (QUS) assesses bone properties through ultrasound wave propagation. QUS parameters, including speed of sound and broadband ultrasound attenuation, correlate with bone density and structure. While QUS does not provide T-scores for osteoporosis diagnosis, it offers radiation-free fracture risk assessment suitable for screening [112].

Finite element analysis (FEA) applied to CT or high-resolution imaging data provides patient-specific estimates of bone strength. FEA models account for bone geometry, density distribution, and loading conditions, potentially providing superior fracture risk prediction compared to BMD alone [113]. Clinical implementation awaits validation and standardization.

### 8.3. Point-of-Care Diagnostics

Portable DXA devices and heel ultrasound systems enable point-of-care bone density assessment. These technologies could facilitate opportunistic screening in primary care settings, potentially improving osteoporosis detection and treatment rates [29]. However, peripheral measurements may not fully reflect spine fracture risk.

Biochemical markers of bone turnover provide dynamic assessment of bone metabolism. C-terminal telopeptide of type I collagen (CTX) and procollagen type I N-terminal propeptide (PINP) are the most widely studied markers. While bone turnover markers show promise for monitoring treatment response and predicting fracture risk, variability and lack of standardization have limited clinical implementation [83,114].

## 9. Novel Therapeutic Approaches

### 9.1. New Pharmacological Agents

Cathepsin K inhibitors represent a novel class of antiresorptive agents that selectively inhibit bone resorption while preserving bone formation. Odanacatib demonstrated significant vertebral and non-OVF reduction in phase III trials. However, development was discontinued due to increased stroke risk, highlighting the challenges of novel drug development [14,115].

### 9.2. Targeted Biological Therapies

Advances in understanding bone biology have identified new therapeutic targets. Inhibitors of dickkopf-1 (DKK-1), an inhibitor of Wnt signaling, promote bone formation and are under investigation. Targeting the activin signaling pathway to promote muscle growth and bone formation represents another promising approach.

Gene therapy approaches for osteoporosis remain largely experimental. Potential strategies include delivery of genes encoding bone morphogenetic proteins or other osteogenic factors. While preclinical studies show promise, clinical translation faces significant challenges [1].

### 9.3. Biomaterial-Based Interventions

Novel bone cements and augmentation materials aim to improve upon conventional polymethylmethacrylate used in vertebroplasty. Calcium phosphate cements offer biocompatibility and gradual remodeling but have lower mechanical strength. Composite materials combining mechanical properties of PMMA with bioactivity of calcium phosphates are under development [14].

### 9.4. Precision Medicine Approaches

Pharmacogenomics may enable personalized osteoporosis treatment. Genetic variants affecting drug metabolism, efficacy, or side effects could guide medication selection. For example, polymorphisms in genes encoding drug targets or metabolizing enzymes may predict bisphosphonate response.

Integration of clinical risk factors, BMD, bone quality parameters, genetic information, and biomarkers into comprehensive risk models could enable more precise fracture risk prediction [113,116]. Machine learning approaches can identify complex patterns and interactions not apparent through traditional statistical methods. Such precision medicine approaches could optimize treatment allocation, targeting intensive therapy to highest-risk individuals while avoiding unnecessary treatment in lower-risk patients.

## 10. Healthcare System Implications

### 10.1. Fracture Liaison Services

Fracture Liaison Services (FLS) represent systematic approaches to secondary fracture prevention. These coordinator-led programs identify patients with fragility fractures, assess osteoporosis, initiate appropriate treatment, and ensure follow-up. FLS implementation has demonstrated significant reductions in subsequent fractures and healthcare costs. A systematic review of FLS programs reported reductions in subsequent fracture rates. The Break Free study demonstrated that comprehensive FLS reduced 90-day subsequent fracture risk by 50% compared to usual care [117]. Cost-effectiveness analyses consistently support FLS implementation, with most studies showing cost savings within 2–3 years [12,14,82,118].

Despite demonstrated efficacy, FLS implementation remains inconsistent globally. Barriers include lack of funding, inadequate staffing, fragmented healthcare systems, and insufficient reimbursement. The International Osteoporosis Foundation has developed standards and guidelines for FLS implementation to facilitate broader adoption.

### 10.2. Secondary Fracture Prevention Programs

Beyond FLS, broader secondary fracture prevention initiatives aim to close the treatment gap [13,119]. The “Own the Bone” program in the United States provides registry and quality improvement tools for orthopedic practices. Similar national and regional initiatives exist in various countries.

Electronic health record (EHR)-based interventions, including clinical decision support systems and automated alerts, can identify patients with fractures and prompt osteoporosis evaluation [117]. Integration of fracture prevention protocols into routine clinical workflows improves treatment rates. However, alert fatigue and competing clinical priorities remain challenges.

Patient education and empowerment are crucial components of secondary prevention. Patients often are unaware of their fracture risk or the availability of effective treatments. Educational interventions, including decision aids and risk communication tools, improve patient knowledge and treatment uptake.

### 10.3. Cost-Effectiveness Considerations

Economic evaluations consistently demonstrate cost-effectiveness of osteoporosis treatment in patients with prevalent fractures [12]. The most cost-effective strategies typically involve treating highest-risk patients, including those with prevalent OVFs. Generic bisphosphonates offer the most favorable cost-effectiveness ratios for most patients.

Anabolic agents, despite higher acquisition costs, may be cost-effective in patients with severe osteoporosis and very high fracture risk [68,82]. Sequential therapy strategies appear cost-effective compared to antiresorptive monotherapy in high-risk populations [12,87]. However, cost-effectiveness varies by healthcare system, pricing structures, and fracture rates.

Universal screening for OVFs remains controversial from a cost-effectiveness perspective. Opportunistic screening using existing imaging may offer better cost-effectiveness ratios than systematic screening programs. Targeted screening of high-risk populations appears most cost-effective.

### 10.4. Policy Recommendations

Healthcare policy interventions are needed to address the growing burden of osteoporotic fractures.

The following recommendations synthesize the existing frameworks of the International Osteoporosis Foundation (IOF) Capture the Fracture program, the Fragility Fracture Network (FFN) minimum standards, the American Society for Bone and Mineral Research (ASBMR) Secondary Fracture Prevention Initiative coalition consensus, and the European Society for Clinical and Economic Aspects of Osteoporosis (ESCEO) policy statements, with additional integration by the authors. They are intended to highlight system-level levers most directly applicable to vertebral-fracture care rather than to constitute new normative guidance [5,14,17,118].

Implementation of systematic secondary fracture prevention programs in all healthcare systems

1. Reimbursement structures that support FLS and coordinator-led models
2. Quality metrics and performance measures for osteoporosis care to drive improvement
3. Integration of bone health into primary care and chronic disease management
4. Public health campaigns to raise awareness about osteoporosis and fracture prevention
5. Research funding prioritization for osteoporosis prevention and treatment

Addressing the global fracture pandemic requires coordinated efforts across clinical medicine, public health, healthcare administration, and policy domains. International collaboration and knowledge sharing can accelerate implementation of effective strategies.

### 10.5. Limitations of the Review

This review has several limitations. First, it was designed as a focused narrative review rather than a systematic review or meta-analysis; therefore, it does not provide exhaustive study identification, formal pooled estimates, or a complete risk-of-bias assessment for every cited study. In keeping with narrative-review conventions, no formal eligibility criteria, dual-reviewer independent screening, or structured data-extraction process were applied; references were selected by the authors based on clinical relevance, methodological rigor, and recency. The PRISMA framework was not used and is not appropriate for the design of this review. Second, the included evidence spans diverse populations, diagnostic definitions, imaging modalities, interventions, and healthcare systems, limiting direct comparison across studies. Third, rapidly evolving areas such as artificial-intelligence-assisted fracture detection, opportunistic CT screening, and sequential anabolic–antiresorptive therapy require further prospective validation before broad implementation. Nevertheless, the authors have endeavored to ensure that the principal clinical claims are supported by representative high-quality evidence drawn from recent international guidelines, systematic reviews, meta-analyses, and major randomized trials.

## 11. Future Perspectives and Conclusions

### 11.1. Unmet Needs

Despite significant advances in OVF diagnosis and management, substantial unmet needs remain [94,107]. The persistent underdiagnosis and undertreatment of OVFs represents the most critical gap [120]. Even when diagnosed, treatment initiation rates remain suboptimal, and long-term adherence is poor.

Better strategies for identifying asymptomatic OVFs are needed. While AI-assisted detection shows promise, widespread implementation requires validation, regulatory approval, and integration into clinical workflows. Standardized reporting protocols for OVFs could reduce underreporting by radiologists.

Current fracture risk assessment tools, while useful, have limited discriminatory ability and may not accurately predict individual patient risk [112]. Development of more precise risk prediction models incorporating multiple risk factors beyond BMD could improve treatment targeting. Biomarkers identifying individuals at imminent fracture risk could enable timely intervention.

Treatment options for patients with contraindications or intolerance to current medications are limited. Novel therapeutic agents with improved efficacy, safety, and tolerability are needed. Oral anabolic agents would substantially improve treatment accessibility and adherence.

Distinguishing levels of clinical readiness: The diagnostic and therapeutic innovations discussed in this review differ markedly in their level of validation, and separating them helps avoid overstating present capability.

Clinically validated technologies: already usable in routine practice, include dual-energy X-ray absorptiometry with Vertebral Fracture Assessment, Genant semiquantitative grading of OVFs, fracture liaison service and other systematic secondary-prevention models, the established antiresorptive and anabolic pharmacotherapies (bisphosphonates, denosumab, teriparatide, abaloparatide, and romosozumab), and regulatory-cleared opportunistic CT tools.

Emerging research concepts: supported by growing but still-maturing evidence and requiring further external validation before routine adoption, include AI-assisted and automated opportunistic fracture detection, bone-quality measures beyond bone mineral density such as the trabecular bone score and finite-element analysis, bone-turnover markers for identifying imminent fracture risk, and optimized sequential or anabolic-first treatment strategies.

Future theoretical applications: presently confined to preclinical or hypothesis-generating work, include orally available anabolic agents, gene therapy, biologics targeting the Wnt inhibitor dickkopf-1 or the activin pathway, pharmacogenomically guided drug selection, and fully autonomous AI interpretation.

### 11.2. Research Priorities

#### Priority Areas for Future Research Include

Epidemiology and Natural History: Better understanding of OVF epidemiology in diverse populations is needed. Longitudinal studies examining factors influencing fracture cascade progression could identify intervention targets. Research on racial and ethnic differences in OVF risk and outcomes could address health disparities [17].

Diagnostic Technologies: Validation and implementation studies of AI-assisted fracture detection are critical. Research on optimal strategies for opportunistic screening could maximize impact while minimizing costs. Development of practical bone quality assessment tools suitable for clinical use represents an important goal.

Therapeutic Advances: Comparative effectiveness research examining different treatment strategies, particularly sequential therapy approaches, would inform clinical practice. Studies identifying optimal treatment duration and monitoring strategies are needed. Research on combination therapies and novel drug delivery systems could improve outcomes.

Implementation Science: Research on effective strategies for implementing secondary fracture prevention programs in diverse healthcare settings is essential. Studies examining barriers and facilitators to treatment adherence could guide interventions. Health services research evaluating different care delivery models would inform optimal program design.

### 11.3. Addressing the Global Fracture Pandemic

The concept of osteoporotic fractures as a global pandemic requires coordinated international response. OVFs, as the most common manifestation and powerful predictors of future fractures, must be central to prevention strategies [4]. A multifaceted approach addressing prevention, early detection, and effective treatment is essential.

Primary prevention through lifestyle modifications, including adequate nutrition, physical activity, fall prevention, and avoidance of smoking and excessive alcohol, can reduce fracture risk across populations [17]. Public health campaigns raising awareness about osteoporosis and fracture prevention are needed.

Secondary prevention, focusing on individuals with established osteoporosis or prevalent fractures, offers the greatest opportunity for near-term impact. Systematic implementation of FLS and secondary prevention programs can substantially reduce the burden of subsequent fractures [121,122]. Healthcare systems must prioritize these proven interventions.

Tertiary prevention, addressing complications and disability following fractures, requires comprehensive rehabilitation programs and multidisciplinary care. Attention to pain management, functional recovery, and psychosocial support improves outcomes and quality of life.

### 11.4. Conclusions

In summary, the central message of this review is that the OVF should be regarded as the clinical entry point to osteoporosis care, mandating a continuous and integrated pathway of diagnosis, fracture-risk stratification, treatment selection, and secondary fracture prevention.

OVFs represent a critical component of the global osteoporotic fracture pandemic, serving as both common manifestations of bone fragility and powerful predictors of future fractures [1]. Despite their prevalence and clinical significance, OVFs remain significantly underdiagnosed and undertreated, representing a major gap in osteoporosis care [25].

The presence of an OVF substantially increases the risk of subsequent vertebral and hip fractures, particularly in the period immediately following the initial fracture [36,38]. This “imminent risk” period represents a critical window for intervention, during which effective treatment can prevent the fracture cascade. Failure to diagnose and treat OVFs allows progression of bone fragility and accumulation of subsequent fractures, with attendant morbidity, mortality, and healthcare costs [123].

Recent advances in diagnostic technologies, particularly AI-assisted fracture detection and opportunistic screening approaches, offer promise for improving OVF detection rates [29,94]. Novel therapeutic approaches, including potent anabolic agents and sequential therapy strategies, provide powerful tools for fracture prevention. Implementation of systematic secondary fracture prevention programs, particularly FLS models, has demonstrated significant success in reducing subsequent fractures [119,122].

Addressing the global burden of OVFs requires action across multiple domains [107]. Healthcare providers must maintain high clinical suspicion for OVFs, utilize appropriate diagnostic imaging, and initiate timely treatment [34]. Healthcare systems must implement systematic approaches to fracture identification and management, including FLS programs and quality improvement initiatives. Policymakers must prioritize bone health, provide adequate funding for prevention programs, and address barriers to osteoporosis care. Researchers must continue developing improved diagnostic and therapeutic approaches while conducting implementation research to optimize care delivery.

The challenge of OVFs and the broader osteoporotic fracture pandemic is substantial but not insurmountable. Evidence-based interventions exist that can dramatically reduce fracture burden. The imperative now is to implement these interventions systematically and at scale, ensuring that all individuals at risk receive appropriate prevention and treatment. Only through such comprehensive efforts can we effectively address the global osteoporotic fracture pandemic and reduce its substantial toll on individuals and societies.

## Data Availability

No new data were created in this study. All data discussed are available in the cited original publications.

## Supplementary Materials

The following supporting information can be downloaded at https://www.mdpi.com/article/10.3390/jcm15135033/s1. Table S1: Synthesis of evidence on the detection and diagnosis of osteoporotic vertebral fractures (OVFs); Table S2: Synthesis of evidence on osteoporotic vertebral fractures (OVFs) as predictors of future fractures and clinical outcomes; Table S3: Synthesis of evidence on the management of osteoporotic vertebral fractures (OVFs).

## Abbreviations

The following abbreviations are used in this manuscript:

AI	Artificial Intelligence
BMD	Bone Mineral Density
BMI	Body Mass Index
CI	Confidence Interval
CT	Computed Tomography
CTX	C-terminal Telopeptide of Type I Collagen
DKK-1	Dickkopf-1
DXA	Dual-Energy X-ray Absorptiometry
EHR	Electronic Health Record
EPOS	European Prospective Osteoporosis Study
FEA	Finite Element Analysis
FLS	Fracture Liaison Service
FRAX	Fracture Risk Assessment Tool
GRADE	Grading of Recommendations, Assessment, Development, and Evaluations
HR-pQCT	High-Resolution Peripheral Quantitative Computed Tomography
IOF	International Osteoporosis Foundation
MRI	Magnetic Resonance Imaging
NSAIDs	Nonsteroidal Anti-inflammatory Drugs
OVF(s)	Osteoporotic Vertebral Fracture(s)
PINP	Procollagen Type I N-terminal Propeptide
PMMA	Polymethylmethacrylate
QUS	Quantitative Ultrasound
RCT	Randomized Controlled Trial
SERMs	Selective Estrogen Receptor Modulators
STIR	Short Tau Inversion Recovery
TBS	Trabecular Bone Score
VFA	Vertebral Fracture Assessment
WHO	World Health Organization
