# Supplementary material for: Osteoporosis: The Renascent Impact of Vertebral Fractures—A Narrative Review of Diagnosis, Risk Stratification, and Integrated Management
Source: J Clin Med. 2026 Jun 28;15(13):5033. doi: 10.3390/jcm15135033 (PMC13362820; doi:10.3390/jcm15135033)
Supplement: Supplementary file 1 [file jcm-15-05033-s001.zip › Table S1_OVF_detection_synthesis.pdf]

Table S1. Synthesis of evidence on the detection and diagnosis of osteoporotic vertebral fractures (OVFs).

Clinical Issue	Representative Evidence	Study Type	Level of Evidence	Strength of Recommendation	Clinical Applicability	Key Finding (Evidence-Driven)	Clinical Implication
OVFs are commonly missed in routine care	Papaioannou et al.; McCabe et al.	Observational (Prospective / Retrospective Cohorts)	2b	—	Applicable in routine outpatient and inpatient care	Observational cohort data demonstrate a persistent underdiagnosis of OVFs, which are independently correlated with increased morbidity, functional decline, and elevated future fracture risk.	Incidental radiographic identification of an OVF warrants immediate reclassification of the patient's risk profile and initiation of secondary fracture prevention protocols.
Targeted VFA indications can improve yield	Kanis et al. (European guidance)	Clinical Practice Guideline	5	Strong	Limited to risk-stratified patients	Evidence-based guidelines strongly support targeted Vertebral Fracture Assessment (VFA) in populations presenting with specific clinical risk factors (e.g., historical height loss >4 cm, kyphosis, glucocorticoid exposure) to optimize diagnostic yield.	Implement risk-stratified case-finding algorithms for VFA rather than indiscriminate population-level screening to ensure cost-effectiveness.
Radiological method affects measured prevalence	Oei et al. (Rotterdam Study)	Population-based Radiographic Cohort (Observational)	2b	—	Applicable in radiological workflows and epidemiological screening	Cohort analyses reveal substantial variability in OVF prevalence depending on the radiological definitions and scoring methodologies	Standardized, validated radiological assessment tools are strictly required to ensure consistent fracture detection and reliable longitudinal

						utilized (e.g., Genant's semi-quantitative criteria).	monitoring.
AI-assisted fracture detection shows promise but remains heterogeneous	Jung et al.; Namireddy et al.	Systematic Review & Meta-Analysis (SR-MA) of Diagnostic Studies	2a	Conditional	Requires specialized AI software and PACS integration	Meta-analyses demonstrate that deep learning and computer vision models achieve high sensitivity and specificity in detecting OVFs, though inter-study heterogeneity exists due to diverse algorithms and imaging modalities.	AI-assisted diagnostic tools may improve detection efficiency and help reduce missed incidental OVFs, but routine clinical implementation requires prospective external validation, standardized reporting, and PACS/EMR workflow integration.

Abbreviations: OVF, osteoporotic vertebral fracture; VFA, vertebral fracture assessment; BMD, bone mineral density; DXA, dual-energy X-ray absorptiometry; FLS, fracture liaison service; RCT, randomized controlled trial; SR-MA, systematic review and meta-analysis; FRAX, Fracture Risk Assessment Tool; PACS, picture archiving and communication system. Levels of evidence follow the Oxford Centre for Evidence-Based Medicine (CEBM): 1a, systematic review of RCTs; 1b, individual RCT; 2a, systematic review of cohort studies; 2b, individual cohort study (including low-quality RCTs); 3, case–control studies; 4, case series; 5, expert opinion, clinical practice guideline, narrative review, or mechanism-based reasoning. For entries graded as Level 5 (clinical practice guidelines or expert consensus), the strength of recommendation reflects clinical consensus rather than high-quality randomized trial evidence. A dash (—) denotes a descriptive or prognostic entry for which a formal strength of recommendation does not apply.
