# Supplementary material for: Osteoporosis: The Renascent Impact of Vertebral Fractures—A Narrative Review of Diagnosis, Risk Stratification, and Integrated Management
Source: J Clin Med. 2026 Jun 28;15(13):5033. doi: 10.3390/jcm15135033 (PMC13362820; doi:10.3390/jcm15135033)
Supplement: Supplementary file 1 [file jcm-15-05033-s001.zip › Table S2_OVF_predictor_synthesis.pdf]

**Table S2.** Synthesis of evidence on osteoporotic vertebral fractures (OVFs) as predictors of future fractures and clinical outcomes.

| Domain | Representative Evidence | Study Type | Level of Evidence | Strength of Recommendation | Clinical Applicability | Key Finding (Evidence-Driven) | Clinical Interpretation |
|---|---|---|---|---|---|---|---|
| Diagnostic significance | Zeytinoglu et al.; Kanis et al. | Clinical Practice Guideline / Narrative Review | 5 | Strong | Applicable in routine clinical practice | The presence of a low-trauma OVF establishes a clinical diagnosis of osteoporosis and identifies the patient as being at high risk of future fracture, independent of densitometric osteoporosis thresholds. | The diagnostic framework must prioritize anatomical fracture events alongside densitometric data to prevent the under-treatment of at-risk patients. |
| Prediction of future OVF | Papaioannou et al.; Zeytinoglu et al. | Prospective Cohort (Observational) | 2b | — | Applicable in routine clinical risk assessment | Longitudinal cohorts consistently demonstrate that a prevalent OVF is a robust, independent predictor of subsequent OVFs, reflecting severe deterioration in bone microarchitecture. | Identification of a recent OVF should be interpreted as an imminent-fracture-risk signal, particularly when fractures are multiple or severe. |
| Prediction of hip and non-OVF | Zeytinoglu et al.; Kanis et al. | SR-MA of Cohort Studies | 2a | — | Applicable in routine clinical risk assessment | Meta-analytic prognostic data show that prevalent OVFs significantly elevate the relative risk for future hip and major non-vertebral fragility fractures. | Spine fractures reflect a systemic skeletal fragility syndrome, necessitating comprehensive systemic pharmacological protection rather than localized structural management. |

| | | | | | | | |
|---|---|---|---|---|---|---|---|
| Fracture cascade and imminent risk | Riccio et al.; Kanis et al.; Kostenuik et al. | SR-MA / Prospective Cohorts | 2a | Strong | Applicable in post-fracture acute and subacute care | The risk of subsequent fracture is acutely elevated (defined as "imminent risk") within the first 1–2 years following an incident OVF, heavily influenced by prior fracture burden. | Recent OVFs warrant prompt anti-osteoporosis therapy, with anabolic-first treatment considered in very-high-risk patients, rather than delayed or deferred reassessment. |
| Number and severity of OVFs | Zeytinoglu et al.; Oei et al. | Prospective Cohort (Observational) | 2b | — | Applicable in radiological reporting and clinical assessment | Observational data indicate that fracture severity (e.g., Genant grades 2–3) and multiplicity exhibit a direct dose-response relationship with the magnitude of future fracture risk. | Radiological severity grading is essential for accurate clinical risk stratification and determining the appropriate aggressiveness of pharmacological regimens. |
| BMD alone is insufficient | Rebolledo et al.; Kostenuik et al.; Allam et al. | SR / Observational Cohorts | 2a | — | Applicable in broad bone health screening | A substantial proportion of incident fragility fractures occur in individuals with osteopenic or normal BMD, highlighting the limitations of DXA in capturing true bone quality and microarchitectural decay. | Validated clinical risk assessment tools (e.g., FRAX) and structural imaging must supplement BMD to comprehensively evaluate skeletal fragility. |
| Functional and rehabilitation implications | Papaioannou et al.; Phruetthiphat et al. | Observational Cohorts | 2b | — | Applicable in geriatric and rehabilitation care | Prevalent OVFs are strongly correlated with impaired physical mobility, chronic pain, and an altered rehabilitation | Post-fracture management must integrate functional rehabilitation and mobility support to mitigate the cascade |

| | | | | | | | |
|---|---|---|---|---|---|---|---|
| | | | | | | trajectory, particularly when concurrent with hip fractures. | of long-term disability and adverse functional outcomes. |
| Treatment intensity | Kanis et al.; Kostenuik et al.; Jin et al. | Clinical Guideline / Expert Consensus | 5 | Strong | Limited to high-risk and very-high-risk patient populations | Evidence-based guidelines endorse risk-stratified treatment algorithms where prior, recent, or multiple OVFs classify patients into very-high-risk categories requiring intensified therapeutic regimens. | Treatment intensity should be tailored to the clinical risk profile (e.g., prioritizing anabolic agents) rather than relying solely on strict densitometric T-score thresholds. |

Abbreviations: OVF, osteoporotic vertebral fracture; VFA, vertebral fracture assessment; BMD, bone mineral density; DXA, dual-energy X-ray absorptiometry; FLS, fracture liaison service; RCT, randomized controlled trial; SR-MA, systematic review and meta-analysis; FRAX, Fracture Risk Assessment Tool; PACS, picture archiving and communication system. Levels of evidence follow the Oxford Centre for Evidence-Based Medicine (CEBM): 1a, systematic review of RCTs; 1b, individual RCT; 2a, systematic review of cohort studies; 2b, individual cohort study (including low-quality RCTs); 3, case–control studies; 4, case series; 5, expert opinion, clinical practice guideline, narrative review, or mechanism-based reasoning. For entries graded as Level 5 (clinical practice guidelines or expert consensus), the strength of recommendation reflects clinical consensus rather than high-quality randomized trial evidence. A dash (—) denotes a descriptive or prognostic entry for which a formal strength of recommendation does not apply.
