# Supplementary material for: Osteoporosis: The Renascent Impact of Vertebral Fractures—A Narrative Review of Diagnosis, Risk Stratification, and Integrated Management
Source: J Clin Med. 2026 Jun 28;15(13):5033. doi: 10.3390/jcm15135033 (PMC13362820; doi:10.3390/jcm15135033)
Supplement: Supplementary file 1 [file jcm-15-05033-s001.zip › Table S3_OVF_management_synthesis.pdf]

Table S3. Synthesis of evidence on the management of osteoporotic vertebral fractures (OVFs).

Management Domain	Representative Evidence	Study Type	Level of Evidence	Strength of Recommendation	Clinical Applicability	Key Finding (Evidence-Driven)	Clinical Implication
Initial conservative management	McCarthy and Davis; Parreira et al.	SR of Clinical Guidelines / Narrative Review	5	Strong	Applicable in primary care and emergency settings	Systematic reviews of guidelines support a short trial of conservative management—including structured analgesia, limited bed rest, and early mobilization—as the first-line therapy for acute OVFs lacking neurological red flags.	Uncomplicated acute OVFs should be managed conservatively initially, with close monitoring for persistent severe pain or progressive spinal deformity.
Vertebral augmentation	Boonen et al.; Zuo et al.; Sun et al.	SR-MA of RCTs	1a	Conditional	Requires surgical expertise; limited to refractory patients	Percutaneous vertebral augmentation may provide faster pain relief and functional recovery in selected acute/subacute painful OVFs refractory to conservative treatment, but evidence is heterogeneous and long-term superiority over optimized conservative care remains debated.	Vertebral augmentation is indicated as a targeted, symptom-modifying intervention for severe cases; it must act as an adjunct to—not a replacement for—pharmacological secondary prevention.
Kyphoplasty vs. vertebroplasty considerations	Li et al.; Movrin; Shawky et al.	Prospective comparative studies	2b	Conditional	Requires specialized interventional settings	Comparative clinical evidence suggests that balloon kyphoplasty may	Procedure selection should be individualized based on fracture

						achieve greater vertebral height restoration and lower cement leakage rates than vertebroplasty, while short-term pain and functional outcomes are broadly comparable.	morphology, acuity, local interventional expertise, and specific patient risk profiles.
Pharmacological secondary prevention	Jin et al.; Saito et al.; Kanis et al.	SR-MA of RCTs	1a	Strong	Applicable to all patients post-OVF	Robust RCT evidence confirms that anti-resorptive (bisphosphonates, denosumab, SERMs) and anabolic agents significantly reduce the incidence of subsequent fractures at both vertebral and non-vertebral sites.	The occurrence of a low-trauma OVF necessitates the immediate initiation of pharmacological therapies, independent of baseline BMD, unless contraindicated.
Very-high-risk and anabolic-first strategy	Kostenuik et al.; Kanis et al.	SR-MA of RCTs	1a	Strong	Limited to very-high-risk patients (e.g., severe/multiple OVFs)	Network meta-analyses of RCTs and recent guidelines indicate that, in specific head-to-head comparisons, initial treatment with osteoanabolic agents followed by antiresorptives may yield greater fracture risk reduction than initial antiresorptive therapy.	An anabolic-first treatment sequence should be preferentially considered for patients presenting with recent, severe, or multiple OVFs to rapidly rebuild bone architecture.
Sequential therapy and denosumab discontinuation	Kanis et al.; Kumar et al.	Observational Cohorts / Guidelines	2b	Strong	Applicable in specialized osteoporosis	Clinical cohort evidence highlights a pronounced rebound increase in bone	A predefined exit strategy incorporating sequential

					management	turnover and an elevated risk of multiple OVFs following denosumab discontinuation without prompt transition to a potent bisphosphonate.	antiresorptive therapy is mandatory prior to the cessation of denosumab to mitigate rebound fracture risk.
Rehabilitation and fall-risk reduction	Riccio et al.; Phruetthiphat et al.; Sun et al.	SR of Observational / Mixed Studies	2a	Conditional	Applicable in multidisciplinary post-acute care	Multimodal rehabilitation programs emphasizing postural correction, trunk and lower-extremity strengthening, balance training, and fall prevention may improve mobility and address modifiable biomechanical and fall-related risk factors.	Comprehensive rehabilitation should be integrated with pharmacological treatment, actively addressing modifiable biomechanical risk factors such as sarcopenia and impaired balance.
Fracture Liaison Service (FLS)	Mitchell; Ozuna et al.; Wu et al.; Lüthje et al.; Kocijan et al.	SR-MA of Cohorts / Health Services	2a	Strong	Applicable at healthcare systems and institutional levels	Systematic reviews of prospective cohorts confirm that multidisciplinary FLS significantly increase rates of BMD testing, initiation of anti-osteoporotic medications, and patient adherence, thereby effectively closing the secondary prevention gap.	Institutions should mandate the implementation of FLS to ensure that both incidental and clinical OVFs routinely translate into coordinated long-term osteoporosis management.

Abbreviations: OVF, osteoporotic vertebral fracture; VFA, vertebral fracture assessment; BMD, bone mineral density; DXA, dual-energy X-ray absorptiometry; FLS, fracture liaison service; RCT, randomized controlled trial; SR-MA, systematic review and meta-analysis; FRAX, Fracture Risk Assessment Tool; PACS, picture archiving and communication system. Levels of evidence follow the Oxford Centre for Evidence-Based Medicine (CEBM): 1a, systematic review of RCTs; 1b, individual RCT; 2a, systematic review of cohort studies; 2b, individual cohort study (including low-quality RCTs); 3, case–control studies; 4, case series; 5, expert opinion, clinical practice guideline, narrative review, or mechanism-based reasoning. For entries graded as Level 5 (clinical practice guidelines or expert consensus), the strength of recommendation reflects clinical consensus rather than high-quality randomized trial evidence. A dash (—) denotes a descriptive or prognostic entry for which a formal strength of recommendation does not apply.
